# Case Reports of *Citrobacter freundii* Infections in Captive Freshwater Species in South Korea

**DOI:** 10.3390/pathogens15080822

**Published:** 2026-08-04

**Authors:** Su-Bhin Jeong, Bo Seong Kim, Min-Young Sohn, Ha-Jeong Son, Chae-Yeong Ji, Ji Hye Jin, Gyoungsik Kang, Chan-Il Park

**Affiliations:** 1 Department of Marine Biology & Aquaculture, Gyeongsang National University, 2 Tongyeonghaeanro, Tongyeong 53064, Republic of Korea; subin8084@naver.com (S.-B.J.); aslhey123@naver.com (C.-Y.J.); 2 Department of Aquatic Life Medicine, College of Ocean Science and Technology, Kunsan National University, Gunsan 54150, Republic of Korea; fishpath@kunsan.ac.kr; 3 Department of Institute of Marine Industry, Gyeongsang National University, 2 Tongyeonghaeanro, Tongyeong 53064, Republic of Korea; thisdancemoment@naver.com (M.-Y.S.); jemma9120@naver.com (H.-J.S.); 4 DaOn Fisheries Diseases Management, Incheon 22379, Republic of Korea; 0327jjh@naver.com; 5 Department of Aquatic Life Medicine, College of Marine Science, Gyeongsang National University, 2 Tongyeonghaeanro, Tongyeong 53064, Republic of Korea

**Keywords:** *Citrobacter freundii*, ocellate river stingray, royal peacock bass, Japanese eel, poison dart frog, histology

## Abstract

*Citrobacter freundii*, an opportunistic Gram-negative bacterium belonging to the Enterobacteriaceae family, is emerging as a significant pathogen in diverse aquatic species, often associated with environmental stress and poor husbandry conditions. This study describes four distinct cases of fatal *C. freundii*-associated lesions in captive freshwater species in South Korea, including an Ocellate river stingray (*Potamotrygon motoro*), a Royal peacock bass (*Cichla intermedia*), a Japanese eel (*Anguilla japonica*), and a Poison dart frog (*Dendrobates leucomelas*). Bacterial DNA was analyzed using 16S rRNA PCR and sequence analysis, and FFPE tissues were additionally examined by PCR for molecular identification. Tissues were examined using routine hematoxylin and eosin (H&E) staining for detailed histopathology. Results: Molecular analysis detected *C. freundii*-associated DNA in the liver, kidney, and intestine of all samples. Histopathology consistently revealed severe bacterial enteritis with epithelial necrosis and abundant intraluminal bacterial aggregates (stingray, frog). Systemic manifestations included severe ulcerative skin lesions (peacock bass), hepatic fatty degeneration (peacock bass, eel), renal tubular degeneration (peacock bass), and prominent hepatic melanomacrophage center activation (frog); however, these findings should be interpreted as lesions observed in association with *C. freundii* detection, as several of these changes are not specific to bacterial infection. To our knowledge, this study provides one of the first comparative histopathological descriptions of naturally occurring *C. freundii*-associated cases across multiple captive freshwater vertebrate species in South Korea. Although bacterial culture, biochemical identification, and experimental infection were not performed, this retrospective investigation identified an association between molecular detection of *C. freundii* and histopathological lesions. Therefore, these findings should be interpreted as supportive diagnostic evidence rather than proof of a definitive causal relationship. These observations highlight the complementary value of histopathological examination alongside molecular detection in the diagnostic evaluation of aquatic animal diseases.

## 1. Introduction

*Citrobacter freundii* is a facultative anaerobic, Gram-negative bacillus belonging to the family Enterobacteriaceae. Although initially recognized as a component of the normal intestinal microbiota in humans and animals, its importance as an opportunistic pathogen has increased since the 1980s following reports describing its association with disease in both terrestrial and aquatic hosts [1,2]. *C. freundii* has been isolated from a wide range of species, such as the Doctor fish (*Garra rufa obtusa*), common carp (*Cyprinus carpio*), zebrafish (*Danio rerio*), giant freshwater prawn (*Macrobrachium rosenbergii*), grass carp (*Ctenopharyngodon idellus*), freshwater stingray (*Potamotrygon motoro*), Japanese eel (*Anguilla japonica*), largemouth bass (*Micropterus salmoides*), crucian carp (*Carassius auratus*), sturgeon, and ornamental fish [3,4,5,6,7,8,9,10,11,12,13,14,15,16,17]. Previous studies have reported that disease outbreaks associated with *C. freundii* are frequently observed under environmental stressors such as high water temperature, high stocking density, and poor water quality, which may increase host susceptibility to opportunistic bacterial colonization or disease [6,7,8,9,10,11,12,13,14,15,16]. Furthermore, *C. freundii* is recognized as an opportunistic zoonotic bacterium that has been associated with urinary tract infections, septicemia, and meningitis in humans, making it a bacterium of concern in both aquaculture and public health [1,2].

In South Korea, diseased individuals of *Garra rufa* were reported to exhibit systemic lesions including skin and fin hemorrhage, gill necrosis, and intra-abdominal bleeding [3]. In China, a strain isolated from the intestine of grass carp was confirmed to be pathogenic in both zebrafish and rodents under experimental conditions [4]. Infection experiments using zebrafish demonstrated that *C. freundii* activates multiple host immune responses, including complement activation, acute-phase responses, mucosal immunity, intestinal-liver immunity, and apoptosis-related pathways [11,13,14]. Recent reports have shown that *C. freundii* exhibits strong pathogenicity in shrimp, with an LD_50_ of 1.1 × 10^6^ CFU/mL, causing inflammation and necrosis in the hepatopancreas, gills, and intestines, and leading to high expression of host immune genes such as *ALF3*, *MyD88*, and *TRAF6* during the acute phase [15]. Furthermore, cases of co-infection have been reported; in common carp, mixed infection with *Aeromonas* spp. and *Plesiomonas shigelloides* resulted in severe clinical signs such as surface hemorrhage, abdominal distension, and gas accumulation in the intestines [16]. Surveys in ornamental fish have indicated that *C. freundii* isolates from various species carry major virulence factors, including the *viaB* capsular antigen and integrons, and show resistance to multiple antibiotics, suggesting difficulties in management [17].

As such, *C. freundii* is closely associated with environmental stressors in aquaculture, such as increased stocking density and poor water quality, and commonly has been detected in association with septicemia, hemorrhage, and tissue necrosis across aquatic animals, including fish, crustaceans, and amphibians. Despite these advances, most previous studies have primarily focused on bacterial isolation, molecular identification, antimicrobial resistance, virulence-associated genes, experimental infection, or host immune responses [4,9,10,11,12,13,14,15,16,17]. Comparatively few studies have systematically described and compared the histopathological characteristics of naturally occurring cases with molecular detection of *C. freundii* across different aquatic vertebrate species. Histopathological examination provides complementary information by allowing direct evaluation of tissue injury, inflammatory responses, and lesion distribution, particularly in retrospective diagnostic investigations where experimental confirmation of causality is not feasible. Furthermore, comprehensive pathological descriptions remain scarce for cartilaginous fishes and captive poison dart frogs, representing important gaps in the current understanding of *C. freundii*-associated disease [5,8,12].

Therefore, the present study describes four retrospective cases in which *C. freundii* was detected by molecular analysis in captive freshwater vertebrates from South Korea. By integrating molecular detection with comparative histopathological examination, this study characterizes the major pathological features observed in each host species and discusses their diagnostic significance in naturally occurring infections.

## 2. Materials and Methods

### 2.1. Sample Collection

Four retrospective diagnostic cases of captive freshwater vertebrates submitted to Gyeongsang National University were included in this study. The cases consisted of one ocellate river stingray (*Potamotrygon motoro*), one royal peacock bass (*Cichla intermedia*), one Japanese eel (*Anguilla japonica*), and one poison dart frog (*Dendrobates leucomelas*). All animals originated from private owners or commercial facilities and were submitted for diagnostic investigation after exhibiting clinical signs. Representative tissues, including the liver, kidney, intestine, and skin (when lesions were present), were collected for molecular and histopathological examinations.

Immediately after necropsy at the collection site, tissue samples were temporarily immersed in 70% ethanol to minimize postmortem decomposition during transportation to the laboratory. Upon arrival at Gyeongsang National University, the tissues were transferred to 10% neutral buffered formalin for re-fixation prior to histopathological processing.

### 2.2. Molecular Biological Analysis

Genomic DNA was extracted individually from all excised organs of the fish and the frog utilizing the AccuPrep^®^ Genomic DNA Extraction Kit (Bioneer, Daejeon, Republic of Korea) in accordance with the manufacturer’s instructions. The extracted genomic DNA samples were stored at −80 °C until further analysis. For general bacterial screening, PCR was performed using the universal primer set 27F/1492R (Table 1). PCR amplification was carried out using a TaKaRa Thermal Cycler Dice Touch TP350 (TaKaRa Bio Inc., Kusatsu, Japan). Subsequently, DNA was extracted from formalin-fixed, paraffin-embedded (FFPE) tissue sections from all fish and frog cases showing histopathological lesions suggestive of bacterial infection, using a commercially available QIAamp DNA FFPE Tissue Kit (Qiagen, Hilden, Germany). The extracted DNA was subjected to 16S rRNA PCR for bacterial species identification (Table 1). The PCR mixture contained 10 μL of Exprime Taq Premix (GeNet Bio, Daejeon, Republic of Korea), 7 μL of distilled water, 1 μL of genomic DNA, and 1 μL each of forward and reverse primers.

Prior to sequence analysis, the PCR amplicons were purified using the QIAquick^®^ Gel Extraction Kit (Qiagen, Hilden, Germany) as per the manufacturer’s protocol. The purified PCR products were then cloned into the pGEM^®^-T Easy Vector (Promega, Madison, WI, USA) and transformed into *Escherichia coli* JM109 competent cells following the standard procedures. After sufficient propagation, plasmid DNA was extracted using the Hybrid-Q™ Plasmid Rapidprep Kit (GeneAll^®^, Seoul, Republic of Korea) and sequenced with the universal M13 primer set. Nucleotide sequence analysis for the identification of *C. freundii* was performed using the Basic Local Alignment Search Tool (BLAST+) version 2.17.0 (National Center for Biotechnology Information, National Library of Medicine, Bethesda, MD, USA) to compare the obtained 16S rRNA sequences with reference sequences available in the NCBI nucleotide database (https://blast.ncbi.nlm.nih.gov/blast; accessed on 22 July 2025) for bacterial species identification, including the molecular identification of *C. freundii*. No bacterial culture or biochemical identification was performed in this study.

### 2.3. Histopathology

After excising the brain, eye, digestive tracts (stomachs and intestines), gills, heart, kidney, liver, and spleen from the fish and the frog, these tissues were initially fixed in 70% ethanol for 24 h and subsequently re-fixed in 10% neutral formalin for an equivalent duration. The tissues then underwent dehydration in a graded series of ethanol (70% to 100%) and clearing with xylene using an automated tissue processor (Leica Biosystems, Wetzlar, Germany), followed by paraffin embedding. Sections were cut at a thickness of 4 μm using an RM2235 Rotary Microtome (Leica Biosystems, Wetzlar, Germany) and stained with hematoxylin and eosin (H&E). Histological sections were examined under a BX50 light microscope (Olympus Corporation, Tokyo, Japan). Representative photomicrographs were captured using a Tech XCam digital camera (Olympus Corporation, Tokyo, Japan) attached to the microscope.

## 3. Results

*Citrobacter freundii* was detected in the liver, kidney, and intestine of all samples tested by molecular analysis following DNA extraction. The same pathogen was also identified in formalin-fixed, paraffin-embedded (FFPE) samples that were histopathologically suspected of *C. freundii* infection.

### 3.1. Case 1: Ocellate River Stingray (Potamotrygon motoro)

**Figure 1:**
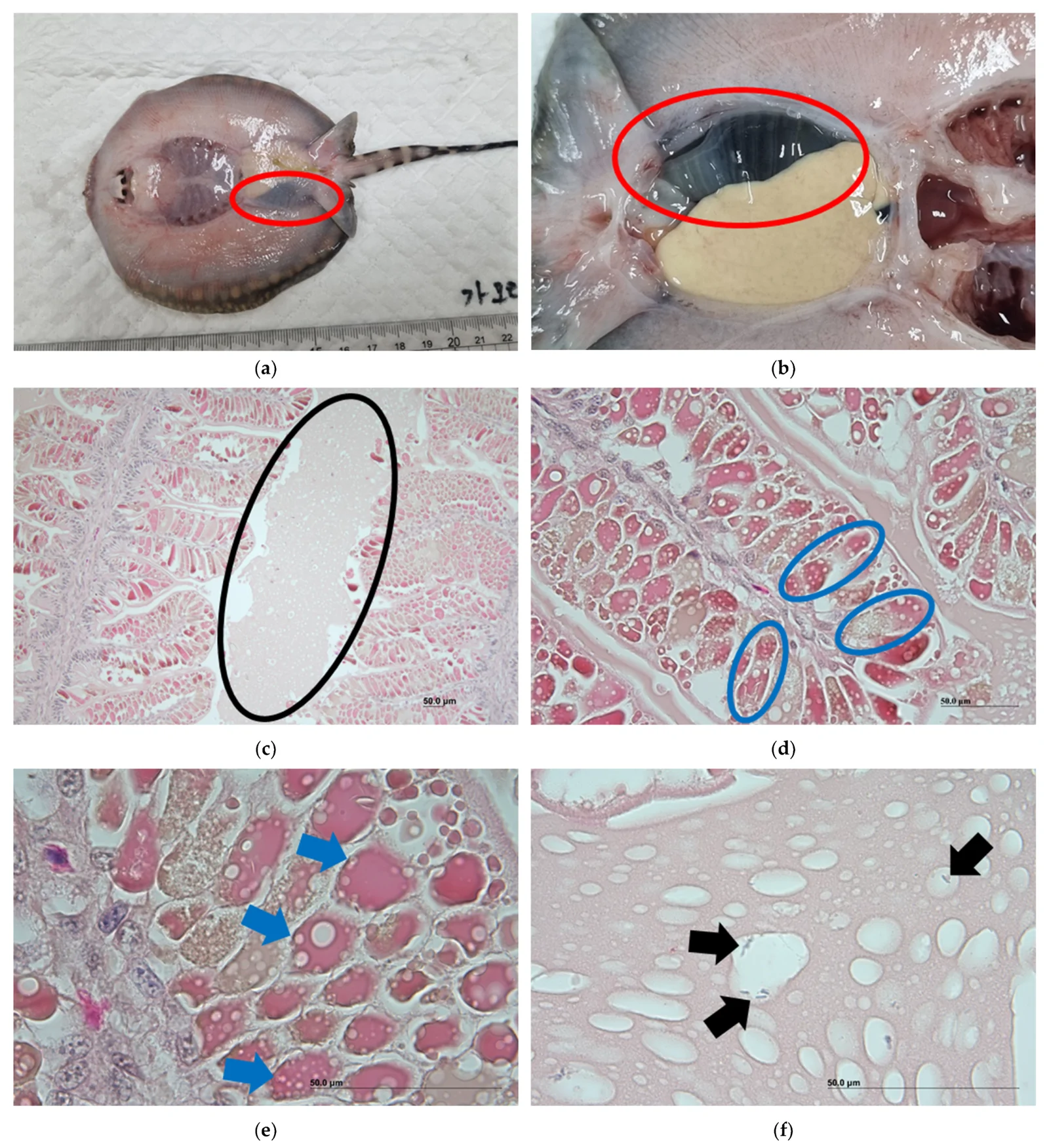
Gross and histopathological findings in an Ocellate river stingray (*Potamotrygon motoro*) with molecular detection of *Citrobacter freundii*. (**a**) Schematic illustration indicating the affected intestinal segment (red circle). (**b**) Gross pathology showing intestinal obstruction with marked luminal distension and accumulation of intraluminal contents (red circle). (**c**) Histopathological section of the intestine showing abundant eosinophilic exudate within the intestinal lumen (black circle). (**d**) Degenerative and necrotic changes of the intestinal epithelium associated with severe inflammatory lesions (blue circles). (**e**) Rod-shaped bacterial aggregates adjacent to the intestinal epithelium (blue arrows), observed in association with the molecular detection of *C. freundii*. (**f**) Numerous intraluminal bacilli within the eosinophilic exudate (black arrows) (H&E; scale bar = 50 μm). These histopathological findings should be interpreted together with the molecular detection results and are not specific to *C. freundii*.

Molecular analysis detected *Citrobacter freundii*-associated DNA in the intestinal tissue. The affected intestinal region is shown in Figure 1a. Infected individuals exhibited pronounced intestinal obstruction, which was clearly observed during abdominal dissection as distension of the intestinal tract and abnormal accumulation of luminal contents (Figure 1b, red circle). Gross examination revealed marked accumulation of intraluminal contents accompanied by severe intestinal distension.

Histopathological examination of the small intestine revealed characteristic lesions, including abundant exudate filling the intestinal lumen between epithelial cells, composed mainly of eosinophilic proteinaceous material and cellular debris (Figure 1c, black circle). Severe pathological alterations of the intestinal epithelium were also evident, with marked cellular degeneration and necrosis accompanied by the structural collapse of the mucosal layer (Figure 1d, blue circle). Under higher magnification, numerous rod-shaped bacterial aggregates were detected within the intestinal mucosa and the accumulated exudate. These bacterial aggregates were frequently located adjacent to the intestinal epithelium (Figure 1e, blue arrows) and were also present within the luminal exudate (Figure 1f, black arrows).

Nine of the ten stingrays maintained in the same aquarium died during the disease outbreak. The examined specimen had a total length of 16.8 cm and a body weight of 38.45 g.

### 3.2. Case 2: Royal Peacock Bass (Cichla intermedia)

**Figure 2:**
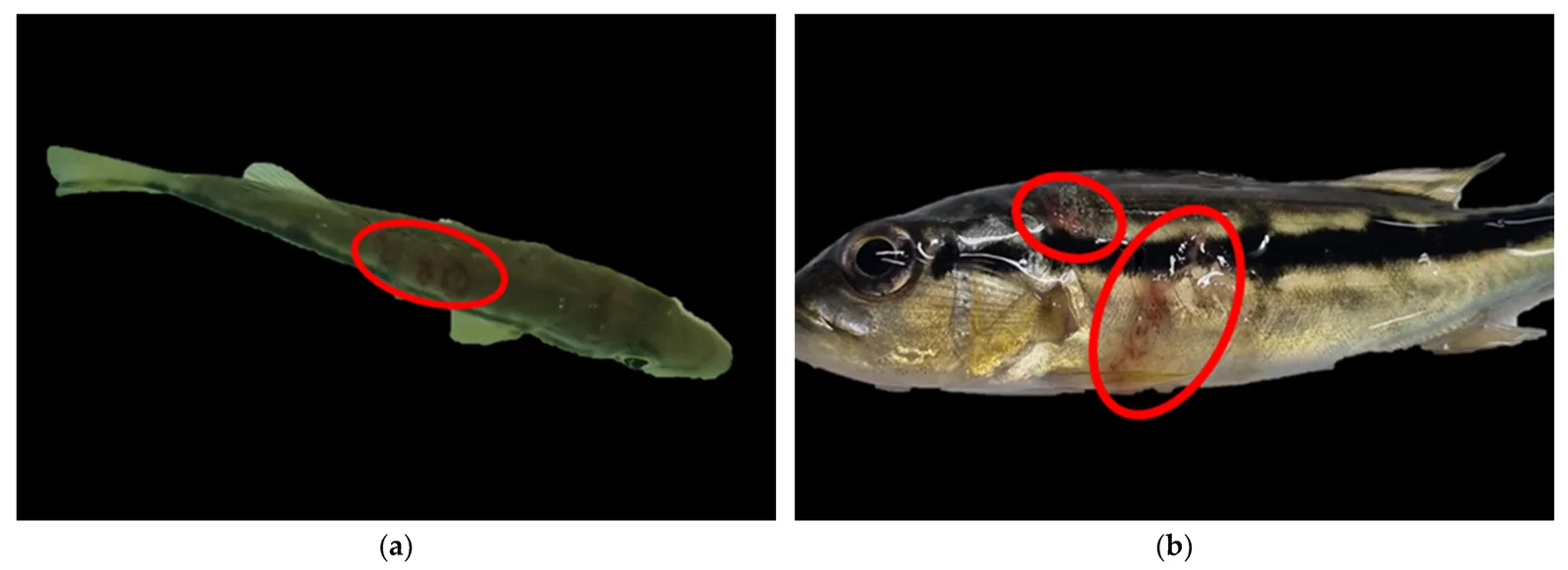

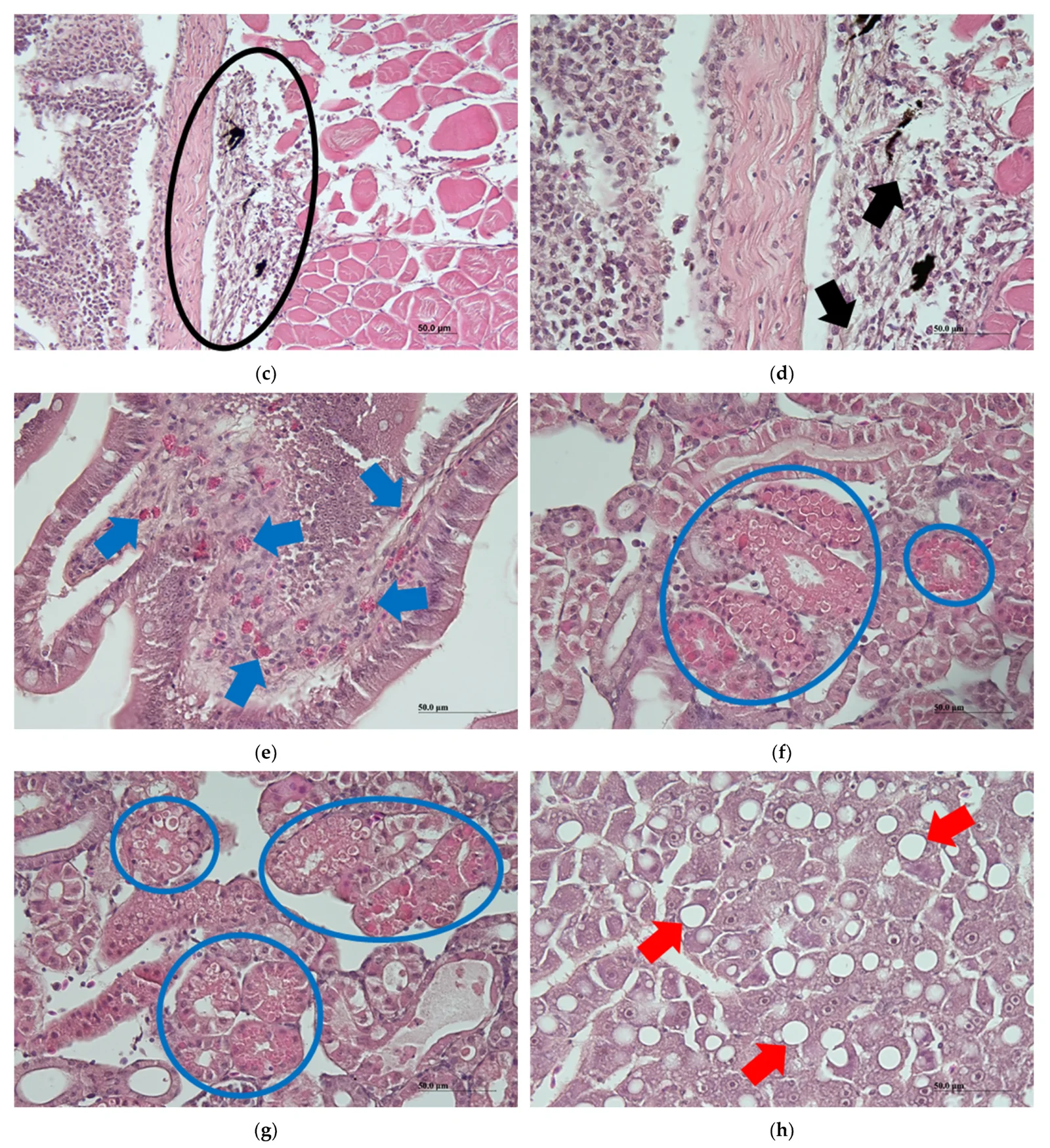
Gross and histopathological findings in a Royal peacock bass (*Cichla intermedia*) with molecular detection of *Citrobacter freundii*. (**a**) Gross image showing ulcerative skin lesions (red circle). (**b**) Schematic illustration indicating the affected skin regions (red circles). (**c**) Histopathological section of the skin showing severe inflammatory cell infiltration with necrotic debris (black circle). (**d**) Rod-shaped bacterial aggregates within the dermis (black arrows), observed in association with the molecular detection of *C. freundii*. (**e**) Infiltration of eosinophilic granular cells (EGCs) in the intestinal submucosa (blue arrows). (**f**,**g**) Renal tubular epithelial degeneration characterized by cytoplasmic vacuolation (blue circles), representing nonspecific histopathological changes. (**h**) Hepatic fatty degeneration characterized by multiple cytoplasmic lipid vacuoles in hepatocytes (red arrows), interpreted as a supportive histopathological finding associated with the molecular detection of *C. freundii* (H&E; scale bar = 50 μm). The histopathological findings should be interpreted together with the molecular detection results and are not specific to *C. freundii*.

The examined Royal peacock bass specimens were of approximately average size, with a mean total length of 7.5 cm and an average body weight of 6.9 g. In infected Royal peacock bass individuals, distinct ulcerative lesions were observed on the skin surface (Figure 2a, red circle), corresponding to the affected regions indicated in the schematic illustration (Figure 2b, red circles). These ulcers involved marked disruption of both the epidermal and dermal layers and appeared grossly as necrotic ulcerations with pronounced peripheral hemorrhagic congestion. Histopathological examination revealed severe infiltration of inflammatory cells within the dermis at the ulcerated sites, accompanied by necrotic tissue debris (Figure 2c, black circle). Numerous rod-shaped bacterial aggregates were detected within the dermal tissue (indicated by black arrows in Figure 2d), suggesting localized bacterial proliferation. In the intestinal tissue, multifocal infiltration of eosinophilic granuloma cells (EGCs) was evident within the submucosal layer (Figure 2e, blue arrows), representing an immunological response to bacterial infection. Renal lesions were characterized by degenerative changes and cytoplasmic vacuolation in the tubular epithelial cells (Figure 2f,g, blue circles). Hepatic sections revealed prominent fatty degeneration of hepatocytes, characterized by the presence of multiple cytoplasmic lipid vacuoles (indicated by red arrows in Figure 2h). These hepatic alterations were considered secondary changes associated with systemic bacterial infection and metabolic disturbance.

### 3.3. Case 3: Japanese Eel (Anguilla japonica)

The examined Japanese eel (*Anguilla japonica*) specimen was of approximately average size, with a total length of 68.2 cm and a body weight of 361.1 g. No remarkable external lesions were observed on gross examination. However, histopathological examination revealed distinct hepatic alterations.

**Figure 3:**
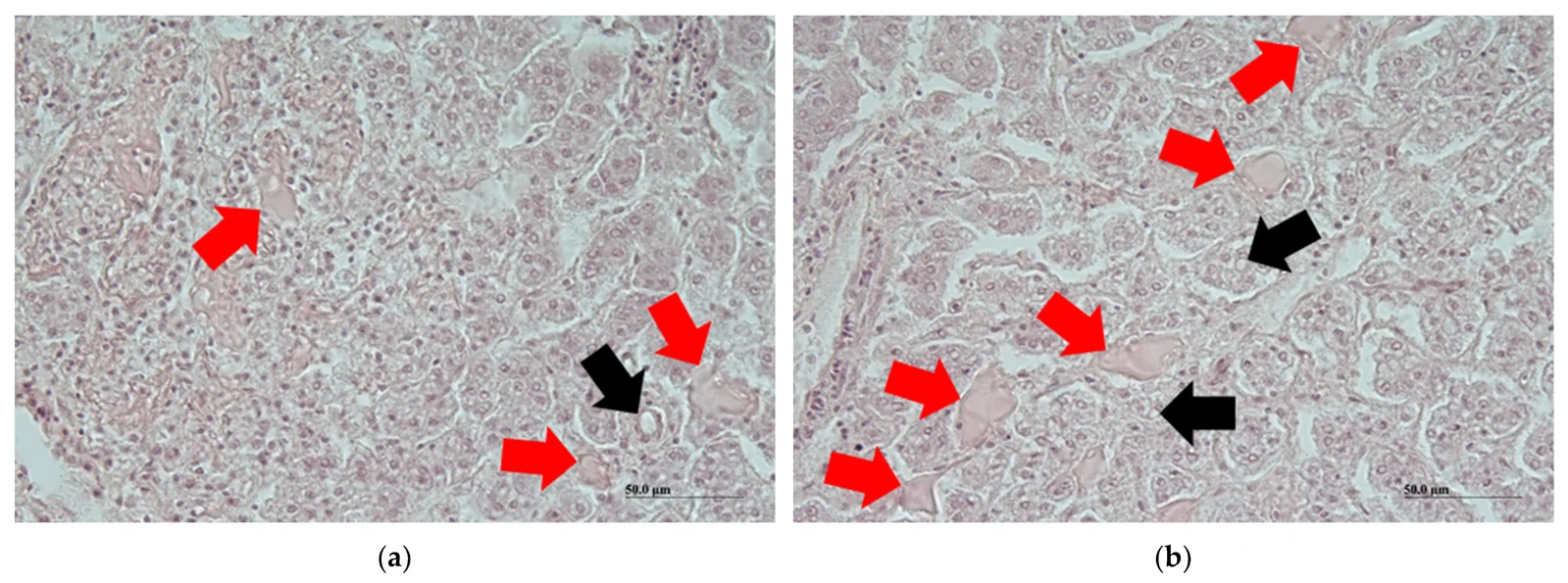
Histopathological findings in the liver of a Japanese eel (*Anguilla japonica*) with molecular detection of *Citrobacter freundii.* (**a**,**b**) Hepatic tissue showing marked fatty degeneration characterized by multiple cytoplasmic lipid vacuoles in hepatocytes (black arrows). Eosinophilic intracytoplasmic materials are also present within hepatocytes (red arrows), representing degenerative and metabolic alterations. The histopathological findings should be interpreted together with the molecular detection results and are not specific to *C. freundii* (H&E; scale bar = 50 μm).

The liver exhibited marked fatty degeneration characterized by numerous cytoplasmic lipid vacuoles within hepatocytes, accompanied by cellular swelling and cytoplasmic degeneration (Figure 3a,b, black arrows). In addition, several hepatocytes contained intensely eosinophilic intracytoplasmic materials (Figure 3a,b, red arrows). These eosinophilic materials were interpreted as proteinaceous cytoplasmic accumulations associated with cellular degeneration and metabolic disturbance. Although these hepatic lesions were observed in association with *C. freundii* detection, they should be interpreted as infection-associated pathological changes rather than pathogen-specific lesions.

### 3.4. Case 4: Poison Dart Forg (Dendrobates leucomelas)

Molecular identification confirmed *Citrobacter freundii* infection in multiple tissues, including the liver, kidney, lung, heart, muscle, skin, and gastrointestinal tract. Notably, *C. freundii* isolates recovered from the hematopoietic organs the liver and kidney showed high genetic similarity to a reference strain previously reported by Pádua et al. (2014) [5]. Sequence analysis revealed 98.38% homology with *C. freundii* deposited in the NCBI database under accession number KC202272, indicating close phylogenetic relatedness to strains isolated in China.

**Figure 4:**
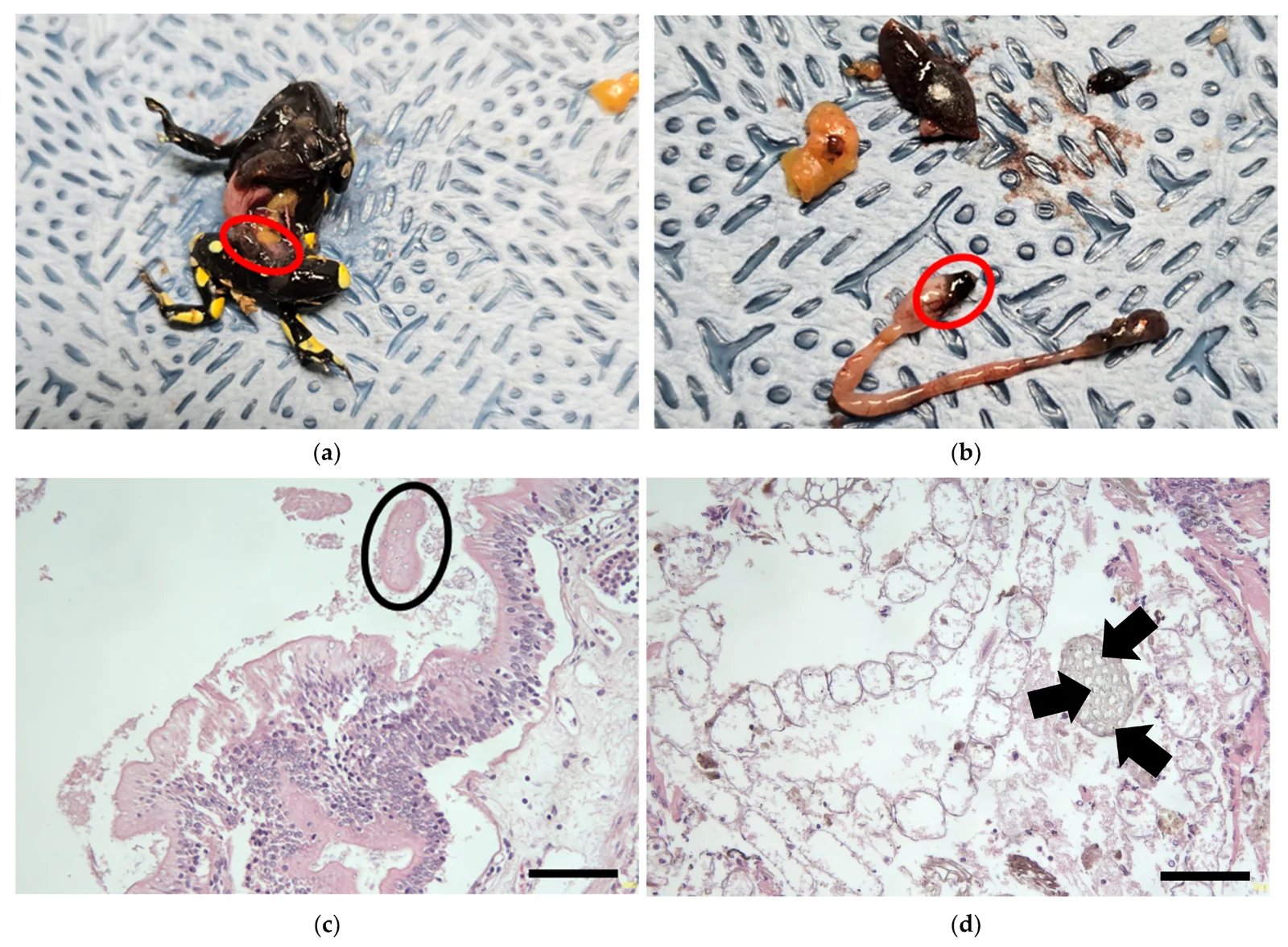

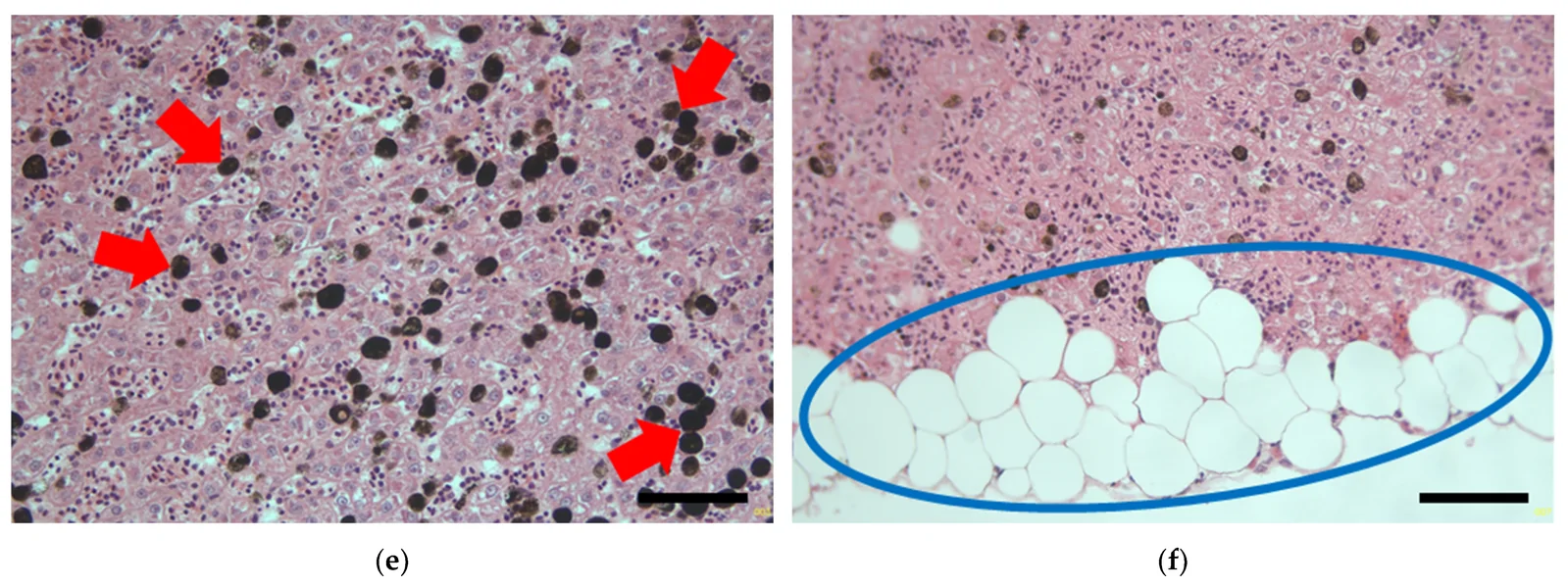
Gross and histopathological findings in a poison dart frog (*Dendrobates leucomelas*) with molecular detection of *Citrobacter freundii*. (**a**,**b**) Necropsy image showing marked accumulation of intraluminal exudate in the intestinal tract (red circle). (**c**) Intestinal mucosa with abundant eosinophilic, proteinaceous exudate filling the lumen; epithelial erosion is present (black oval). (**d**) Higher magnification of the intestinal lumen revealing numerous rod-shaped bacteria embedded within the exudate and along the mucosal surface (black arrows), observed in association with the molecular detection of *C. freundii*. (**e**) Liver with prominent melanin pigmentation represented by increased melanomacrophage centers within the hepatic parenchyma (red arrows). (**f**) Liver showing conspicuous subcapsular accumulation of adipose tissue beneath the hepatic capsule (blue oval), representing a nonspecific histopathological finding (H&E; scale bar = 50 μm). The histopathological findings should be interpreted together with the molecular detection results and are not specific to *C. freundii*.

Anatomical examination of the poison dart frog (*Dendrobates leucomelas*) revealed extensive accumulation of mucoid and proteinaceous exudate within the intestinal lumen, similar to the lesion observed in Case 1 involving *Potamotrygon motoro* (Figure 4a,b, red circle). The accumulation of intraluminal material appeared to restrict intestinal content movement. Histopathological observation showed partial erosion of the intestinal mucosa, with the lumen filled with abundant eosinophilic, proteinaceous exudate admixed with necrotic debris and exfoliated epithelial cells (Figure 4c, black oval). At higher magnification, numerous rod-shaped bacteria were detected within the exudate and along the mucosal surface (Figure 4d, black arrows), consistent with bacterial enteritis. In the liver, a marked increase in melanin-containing cells was observed throughout the hepatic parenchyma, appearing both scattered and clustered, consistent with activation of melanomacrophage centers (MMCs), a common histopathological response to chronic inflammation or infectious conditions in amphibians (Figure 4e, red arrows). Additionally, prominent subcapsular accumulation of adipose tissue was observed beneath the hepatic capsule (Figure 4f, blue oval), consistent with subcapsular fat deposition or expansion of adjacent fat bodies.

Collectively, these findings demonstrate severe intestinal lesions accompanied by hepatic MMC activation and subcapsular lipid accumulation in a case with molecular detection of *Citrobacter freundii*. These pathological findings are considered to be associated with systemic infection, although they should be interpreted in conjunction with the molecular detection results and overall clinicopathological findings.

The examined poison dart frog specimen measured 4.17 cm in snout–vent length and weighed 7.6 g. Collectively, the molecular detection of *C. freundii* and the accompanying histopathological lesions provide supportive diagnostic findings; however, because bacterial culture and experimental infection were not performed, these observations should be interpreted as an association rather than evidence of a definitive causal relationship.

## 4. Discussion

*Citrobacter freundii* is an opportunistic pathogen widely distributed in aquatic environments and is increasingly recognized as an important pathogen associated with disease outbreaks in various aquatic species, including fish, crustaceans, and amphibians. Since the first reports describing *C. freundii*-associated disease in common carp (*Cyprinus carpio*) [16] and Doctor fish (*Garra rufa obtusa*) [3], the bacterium has been associated with hemorrhagic enteritis and systemic septicemia under environmental stress conditions such as elevated temperature, hypoxia, and high rearing density [9]. Subsequent cases reported in grass carp, catfish, and ornamental fish from China, Brazil, and Europe have confirmed that *C. freundii* possesses a broad host range and high environmental adaptability as an opportunistic aquatic bacterium [5,6,7,8]. In the present study, consistent histopathological alterations, including intestinal obstruction, epithelial necrosis, hepatic fatty degeneration, and splenic lesions, were observed, and these findings were generally consistent with previous reports of naturally occurring *C. freundii*-associated cases. In particular, necrotic lesions in the liver and intestines were compatible with pathological changes previously described in septicemic bacterial infections [1]. Moreover, in amphibians, accumulation of proteinaceous exudate in the intestine and proliferation of melanomacrophage centers were observed together with molecular detection of *C. freundii*, suggesting an inflammatory response potentially associated with the presence of the bacterium. Although these lesions are consistent with previously reported findings, they are not specific to *C. freundii* and should therefore be interpreted in conjunction with histopathological and molecular findings rather than as definitive evidence of pathogen-specific lesions. Furthermore, because the present study was based on retrospective diagnostic submissions, the observed pathological findings should be interpreted as being associated with *C. freundii* detection rather than as definitive evidence of pathogen-specific lesion development. Although bacteriological isolation and molecular detection supported the diagnosis, experimental infection studies were not performed to establish a direct causal relationship in each host species.

Host susceptibility to *C. freundii* has been reported to vary markedly depending on species and developmental stage. In experimental infections using zebrafish (*Danio rerio*), a significant upregulation of genes related to complement activation and apoptosis was observed, indicating a strong innate immune response. Similarly, in the giant freshwater prawn (*Macrobrachium rosenbergii*), expression levels of immune-related genes such as *ALF3*, *MyD88*, *TRAF6*, and *TNF* increased sharply during the acute phase of infection, suggesting activation of systemic immune defense mechanisms [15]. In the sturgeon infection model, hematological alterations—including leukocytosis, erythropenia, and elevated activities of serum enzymes such as ALT, LDH, and ACP—were detected, demonstrating that acute infection is accompanied by systemic inflammation and oxidative stress responses [13,14]. Collectively, these previous experimental studies demonstrate that *C. freundii* can induce innate immune and oxidative stress responses under controlled experimental conditions in diverse aquatic species. Although the present study did not evaluate host immune responses or experimentally confirm pathogenicity, the histopathological findings observed in the retrospective cases were generally comparable to lesions reported in these experimental studies, further supporting the broad host range and opportunistic characteristics of *C. freundii* in aquatic animals.

In naturally occurring cases, *C. freundii* was more frequently detected as part of mixed infections rather than as a single pathogen, commonly in association with *Aeromonas* spp. or *Plesiomonas shigelloides* [16]. These co-infections were often accompanied by severe clinical manifestations such as surface hemorrhage and intestinal gas accumulation, which were closely related to deteriorating environmental factors, including high stocking density, reduced dissolved oxygen levels, and temperature fluctuations [1,10,16]. Moreover, *C. freundii* has been reported to persist within tank sediments and intestinal microbial communities, potentially increasing the likelihood of opportunistic colonization or disease under environmental stress conditions [4,13,14]. Recently, multidrug-resistant (MDR) strains have been increasingly reported worldwide. In ornamental fish isolated from Poland, 100% resistance to ampicillin and high levels of resistance to tetracyclines and quinolones were observed [17]. In addition, the presence of the *viaB* capsular antigen and class 1 integron genes suggests the potential for enhanced virulence and horizontal gene transfer. Isolates from Asian regions also carried adhesion- and toxin-related genes such as *ompX*, *ureG*, and *cfa1/2*, which may contribute to bacterial colonization and tissue invasion [10,11]. Although antimicrobial susceptibility and virulence-associated genes were not investigated in the present study, these previous findings indicate that molecular detection of *C. freundii* in diseased aquatic animals should be interpreted with consideration of its pathogenic potential and increasing antimicrobial resistance. Together, these findings highlight the pathogenic potential and antimicrobial resistance of *C. freundii*, emphasizing the importance of continuous surveillance and appropriate disease management in aquaculture systems [11,15,17].

It should be noted that molecular identification of *Citrobacter freundii* in the present study was based on 16S rRNA sequence analysis of PCR amplicons obtained from FFPE tissues rather than on bacterial culture or biochemical identification. The ocellate river stingray case showed extensive pathological lesions and mortality under captive conditions [8], whereas the poison dart frog case represents a rare and diagnostically important instance of *C. freundii*-associated disease in an amphibian host. These cases expand the currently available information regarding the host range, tissue distribution, and pathological features associated with molecular detection of *C. freundii* across phylogenetically distinct aquatic species. In contrast, the pathological manifestations observed in the peacock bass and Japanese eel were highly consistent with previously reported *C. freundii*-associated lesions in teleost fishes [5,6,7,9,10], and the combined molecular and histopathological findings were considered supportive of the diagnosis. Accordingly, gross pathological, histopathological, and molecular findings were interpreted together in all cases to provide diagnostic evidence, while acknowledging that molecular detection alone does not establish a direct causal relationship between *C. freundii* and the observed lesions.

*C. freundii* is recognized as a zoonotic pathogen that causes urinary tract infections, septicemia, and meningitis not only in fish and crustaceans, highlighting its potential public health significance [2,12,17]. With the globalization of the ornamental fish trade and the international exchange of aquatic organisms, environmental stress and cross-border distribution have become major factors contributing to the dissemination of this pathogen [12,17]. In conclusion, *C. freundii* is an emerging opportunistic pathogen associated with disease in a wide range of aquatic vertebrates and invertebrates [1,5,8,9,10,11,12,15]. Previous studies have shown that experimental *C. freundii* infection may be accompanied by hemorrhagic enteritis, hepatocellular and renal lesions, and innate immune and oxidative stress responses [5,7,11,13,14,15], while exhibiting high levels of multidrug resistance and environmental adaptability, which may complicate disease management in aquaculture systems [11,15,17]. In the present study, molecular detection of *C. freundii* together with histopathological examination provided supportive diagnostic evidence in retrospective clinical cases; however, because bacterial culture and experimental infection were not performed, a direct causal relationship between *C. freundii* and the observed lesions could not be confirmed. Therefore, preventive measures focusing on broodstock and seed management, maintenance of water hygiene, and reduction of antibiotic use are essential. Further studies combining bacterial isolation, experimental infection, molecular characterization, and investigations of host–pathogen interactions will be crucial for improving the understanding of *C. freundii*-associated disease and for developing effective preventive strategies and potential vaccines.

Importantly, the present study expands the currently available pathological descriptions of naturally occurring *C. freundii*-associated cases in captive freshwater animals in South Korea. Diagnosis was supported by an integrative approach combining molecular detection based on 16S rRNA sequence analysis and histopathological examination of multiple organs (intestine, liver, and kidney). The pathological findings observed in these cases were generally consistent with previous reports and provide additional reference data for diagnostic investigations of *C. freundii*-associated disease in aquatic animals. Furthermore, the combined application of molecular detection and histopathological evaluation may facilitate future case investigations, differential diagnosis, and disease surveillance in aquaculture settings. The present study also has several limitations. Because only four retrospective cases representing different aquatic species were included, caution is warranted when extrapolating these findings to other host species or production systems. In addition, because bacterial culture, biochemical identification, antimicrobial susceptibility testing, and experimental infection were not performed, the molecular detection of *C. freundii* should be interpreted as supportive diagnostic evidence rather than definitive proof of causation. Nevertheless, the integration of molecular detection and histopathological examination provides valuable diagnostic evidence and expands the currently available pathological descriptions of naturally occurring *C. freundii*-associated cases in captive freshwater animals.

## 5. Conclusions

This study describes four retrospective cases with molecular detection of *Citrobacter freundii* in captive freshwater animals in South Korea and highlights the complementary value of combining molecular detection with histopathological examination during diagnostic investigations. The findings provide additional pathological reference data for *C. freundii*-associated cases in captive freshwater animals and broaden the currently available information regarding their pathological characteristics and host distribution. Although bacterial culture and experimental infection were not performed, the combined interpretation of molecular and histopathological findings provides supportive diagnostic evidence for retrospective case investigations. Improved husbandry practices and routine disease surveillance may contribute to reducing the risk of future disease outbreaks in captive populations.

## Figures and Tables

**Table 1:** Primers and PCR conditions used in this study.

Target	Primer	Sequences (5′-3′)	Condition	References
General bacteria	27F	AGAGTTTGATCMTGGCTCAG	95 °C for 3 min, (95 °C for 30 s, 55 °C for 30 s, 72 °C for 30 s) ×35 cycles, 72 °C for 7 min	[18]
	1492R	TACGGYTACCTTGTTACGACTT		

## Data Availability

The original contributions presented in this study are included in the article. Further inquiries can be directed to the corresponding author.
